# How to deal with 2019 novel coronavirus (COVID-19): Public health practices from the Centers for Disease Control and Prevention in Zhanggong District, Ganzhou City, China

**DOI:** 10.1017/ice.2020.110

**Published:** 2020-04-06

**Authors:** Peisheng Xiong, Kai Xu, Guang Xiao

**Affiliations:** Zhanggong District Center for Disease Control and Prevention, Ganzhou, Jiangxi, PR China

*To the Editor—*Since the first case of novel coronavirus (COVID-19) appeared in Wuhan, China, in December 2019, it spread quickly across China and has now been detected in >200 counties around the world. By April 10, 2020, the cumulative number of confirmed cases reached 83,305 and deaths reached 3,345 in China. The number of reported cases has also increased rapidly in many other countries. To date, >1,400,000 cases have been confirmed and 80,000 deaths have occurred outside China. Now, the global number of reported cases of COVID-19 has surpassed 1,500,000.^1^

Zhanggong District is the central area of Ganzhou City, Jiangxi Province, China. It covers an area of 425.5 km^2^ and has a total population of 620,000. Zhanggong faced a severe public health challenge in the COVID-19 emergency. The Zhanggong District exports a large amount of adult labor to other places for employment, including Wuhan City, Hubei Province. During the Spring Festival, the people who live and work in other cities (including Wuhan) return to the Zhanggong District to celebrate the festival with their families. After the first case was diagnosed on January 23, 2020, the number of COVID-19 pneumonia patients in the Zhanggong District increased substantially after the Spring Festival. To prevent the situation from getting worse, the Zhanggong District government set up a joint prevention and control mechanism to stop the spread of the disease, composed of the following departments: (1) Ganzhou Municipal Party School, (2) Ganzhou Jude Mountain Villa, (3) Shahe Branch of Ganzhou Municipal Hospital, (4) Fifth People’s Hospital of Ganzhou, (5) Ganzhou Wulong Hakka Style Garden, and (6) Ganzhou Center for Disease Control and Prevention.

When the emergency command received the suspected patient information from the fever clinic or the WeChat Mini Program client, the suspected patient was sent to the Ganzhou Municipal Hospital for physical examination. The Zhanggong District CDC was notified to conduct an epidemiological investigation simultaneously. They sent the throat and nasal test results, as well as the blood samples collected from the Ganzhou Municipal Hospital. The blood samples underwent polymerase chain reaction (PCR) testing, and if the PCR test was positive, the patient was sent to Ganzhou Fifth People’s Hospital for isolation treatment. If the test result was negative, the suspected patient was sent to isolation locations (Ganzhou Wulong Hakka Style Garden, Ganzhou Municipal Party School, Ganzhou Jude Mountain Villa and Shahe Branch of Ganzhou Municipal Hospital) for 14 days of medical isolation observation, during which time the suspected patient was screened many times until the quarantine period ended (Fig. 1a). The time from receiving the suspected patient’s information to the initial diagnosis was <24 hours. Using rigorous screening, we identified a total of 28 COVID-19–positive patients and >600 close contacts.

**Fig. 1.**
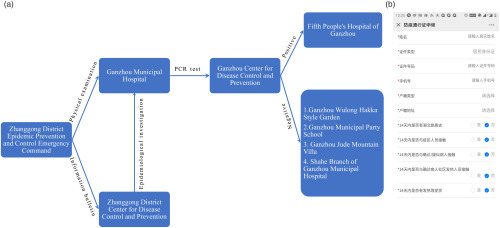
(a) Suspected patient treatment process, the entire treatment process does not exceed 24 hours. (b) WeChat Mini Program Client. Note. PCR, polymerase chain reaction.

In addition, the local government of Zhanggong District decided to block the highway during the epidemic and advised the public to stay at home. At the same time, the government prohibited all stores in the market from opening, except drug stores and malls. All members of the public were encouraged to record their health status on the WeChat Mini Program client, including whether they have been to Wuhan, Hubei, whether they had had close contact with diagnosed patients, and their body temperature (Fig. 1b).

We believe that the key point to handling the COVID-19 epidemic is rapid action. The sooner patients and their close contacts are identified, the more likely that effective control of the epidemic can be achieved. We hope that the lessons we learned from this outbreak will help other regions in similar situations.

## References

[1] Novel coronavirus (2019-nCoV)-situation report-81. World Health Organization website. https://www.who.int/docs/default-source/coronaviruse/situation-reports/20200410-sitrep-81-covid-19.pdf?sfvrsn=ca96eb84_2. Published April 10, 2020. Accessed April 11, 2020.

